# Focal Status and Sub-Ependymal Tumor as Features of the First Presentation in a Child With Tuberous Sclerosis

**DOI:** 10.7759/cureus.11816

**Published:** 2020-12-01

**Authors:** Kavinda Dayasiri, Vijayakumary Thadchanamoorthy, Kaushika Thudugala, Maheshaka Wijewardana

**Affiliations:** 1 Paediatrics, Base Hospital, Mahaoya, LKA; 2 Clinical Sciences Department, Eastern University, Faculty of Health Care Sciences, Batticaloa, LKA; 3 Paediatrics, District General Hospital Ampara, Ampara, LKA

**Keywords:** sub-ependymal astrocytoma, tuberous sclerosis

## Abstract

Tuberous sclerosis (TS) is an autosomal dominant inherited disorder that affects multiple organ systems. Usually, children with TS present either with neurocutaneous stigmata or seizures during the early years of life. The mortality and morbidity are related to refractory epilepsy, giant cell astrocytoma and related complications, and multiple angiomyolipomas. The authors have reported an eleven-year-old child in whom focal status and sub-ependymal tumor were the features of the first presentation of tuberous sclerosis. The report further highlights the importance of early identification of cutaneous features by primary care providers and parents to enable early comprehensive multi-disciplinary management.

## Introduction

Tuberous sclerosis is an autosomal dominant neurocutaneous syndrome characterized by the triad of epilepsy, mental retardation, and sebaceous adenoma [1, 2]. It has a population prevalence of 1:20,000 [1]. Most children with tuberous sclerosis complex are diagnosed early in life following either presenting with seizures or based on neurocutaneous stigmata [3]. The majority of cases of tuberous sclerosis occur due to pathogenic mutations in TSC2 (encodes for tuberin) whilst TSC1 (encodes for hamartin) and other rare mutations account for remaining cases [4]. The disease is known to affect multiple organ systems, including the central nervous system, heart, lungs, kidneys, eyes, teeth, skin, smooth muscle, and adipose tissue [5]. We report a child who presented with focal status associated with sub-ependymal tumor as the first presentation of tuberous sclerosis.

## Case presentation

An eleven-year-old boy from a remote village presented with focal status epilepticus. The seizure initially started with the head, mouth, and both eyes deviating to the left side and followed by generalized tonic movements. The seizure terminated at 30 minutes following intravenous benzodiazepines given in the emergency department. Clinic records were not available on admission, and to caregivers’ knowledge, he had been previously in good health. However, his parents had noticed hyperpigmented patches and small dark bumps over the face for the last five years, which had been progressively increasing in number. Although he had been offered follow-up in the pediatric clinic after viral illness two years ago, he discontinued, and no evaluation could be performed. The specific inquiry also revealed gross and fine motor developmental delay and moderate learning difficulties. There was no family history of brain tumors or similar skin lesions. His mother had acquired lower limb paralysis and was diagnosed with Fisher-Bickerstaff syndrome, which is a rare inflammatory disorder of the central and certain extend to the peripheral nervous system.

**Figure 1:**
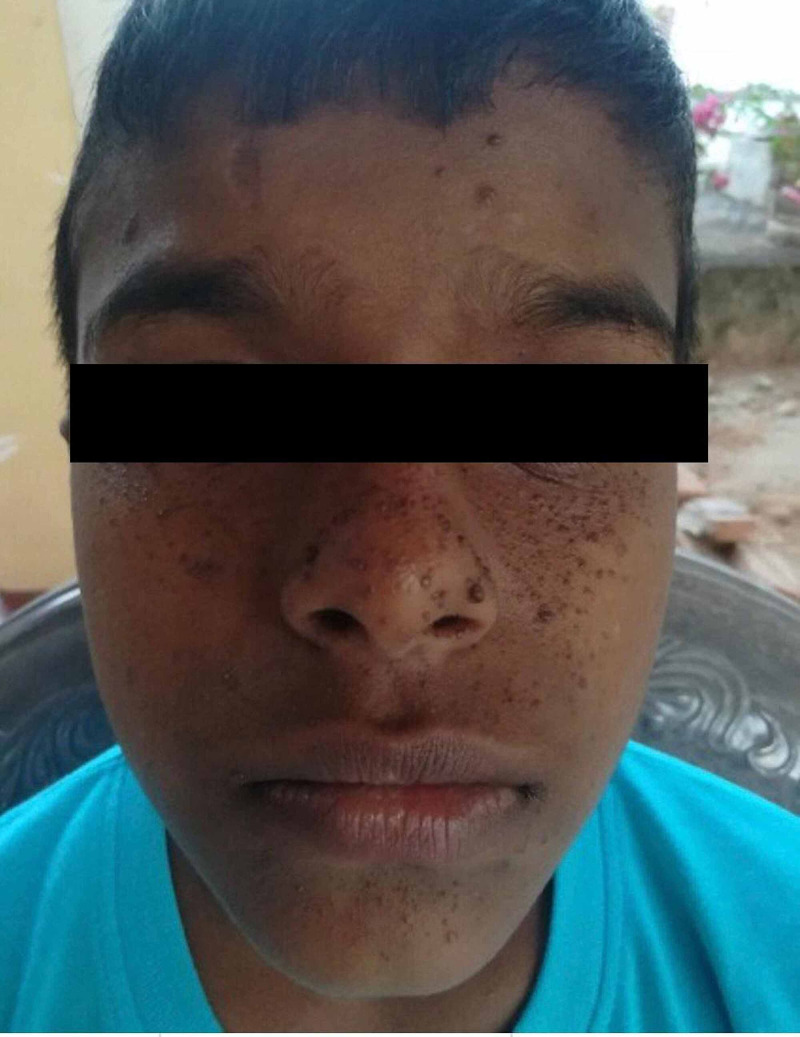
Facial angiofibromas

**Figure 2:**
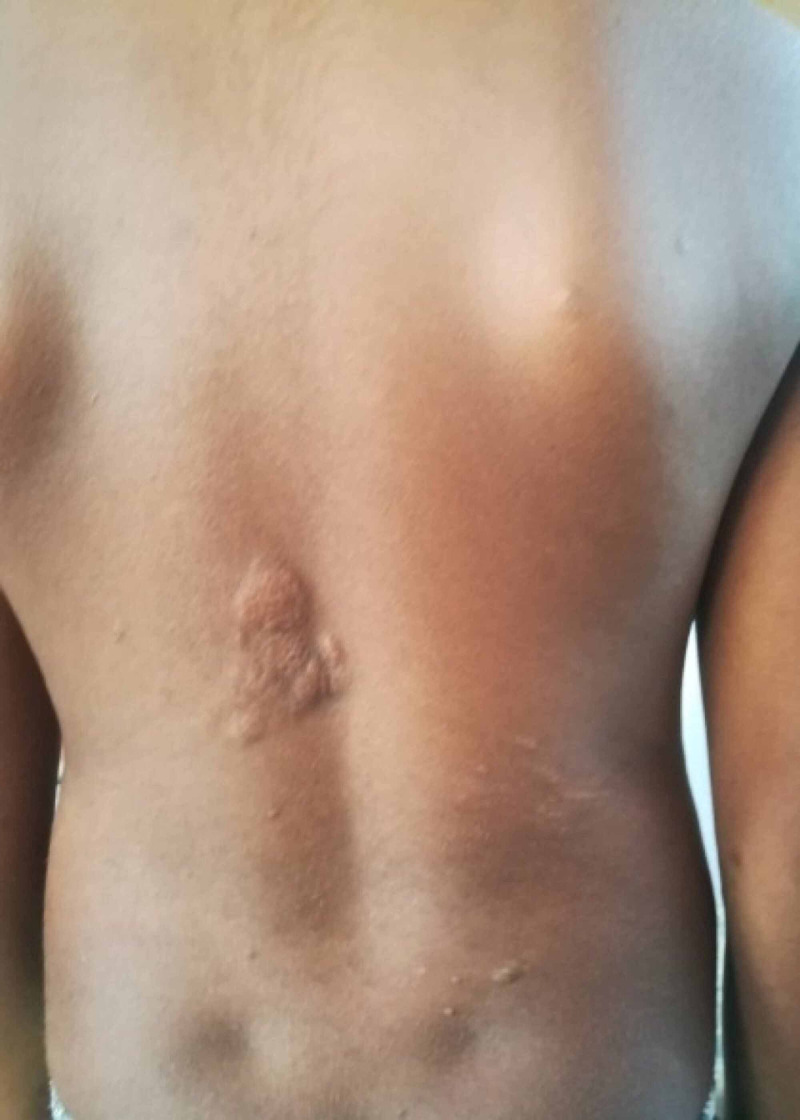
Shagreen patches

**Figure 3:**
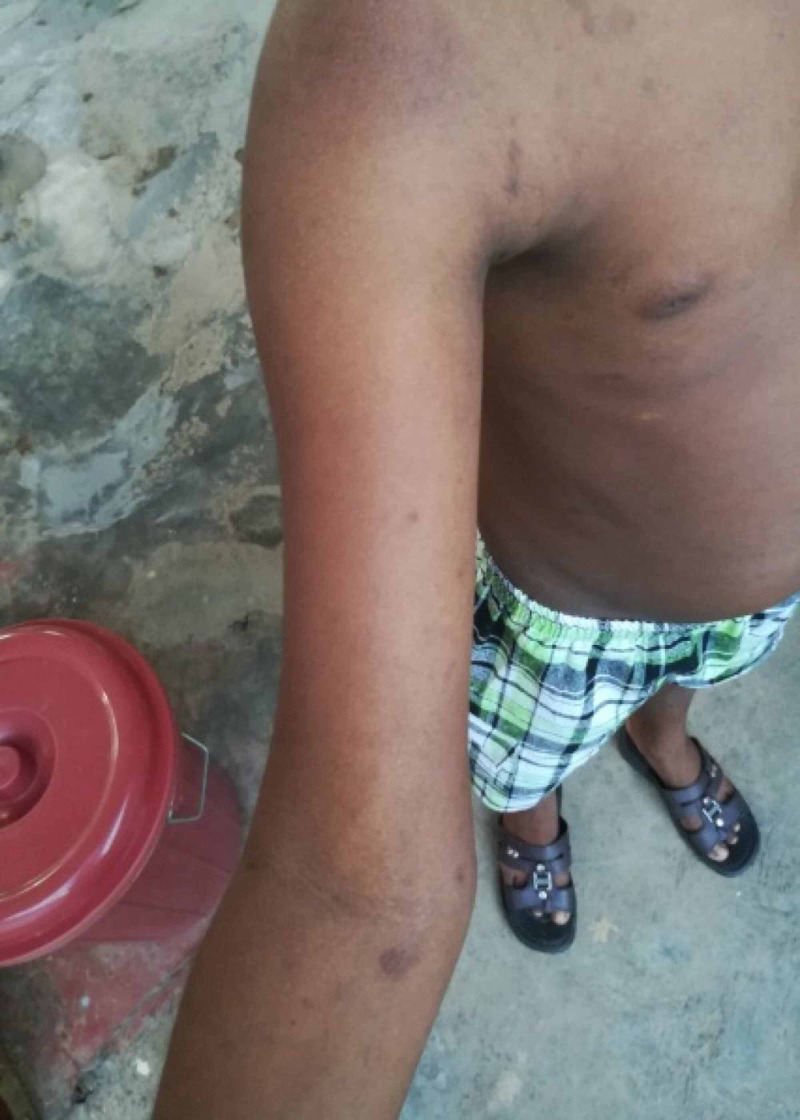
Multiple café-au-lait patches

Physical examination revealed facial angiofibromas, multiple café-au-lait patches, ash leaf macules, and shagreen patches. Neurological examination of cranial nerves, upper and lower limbs was unremarkable. The possibility of giant cell astrocytoma was suspected, and the child was transferred for urgent contrast-enhanced CT brain. Figures 1-3 show facial angiofibromas, shagreen patches, and multiple café-au-lait patches in the reported child respectively.

**Figure 4:**
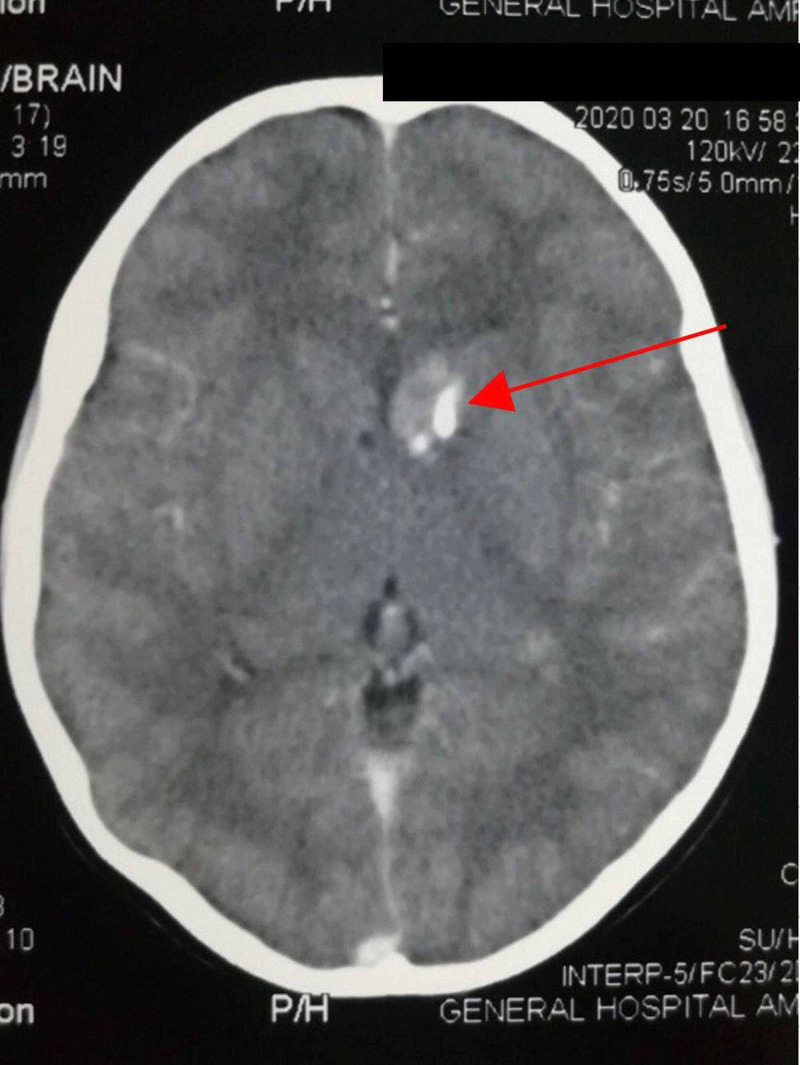
Contrast-enhanced CT showing 12 mm hyperdense mass adjacent to the left anterior horn of the lateral ventricle

**Figure 5:**
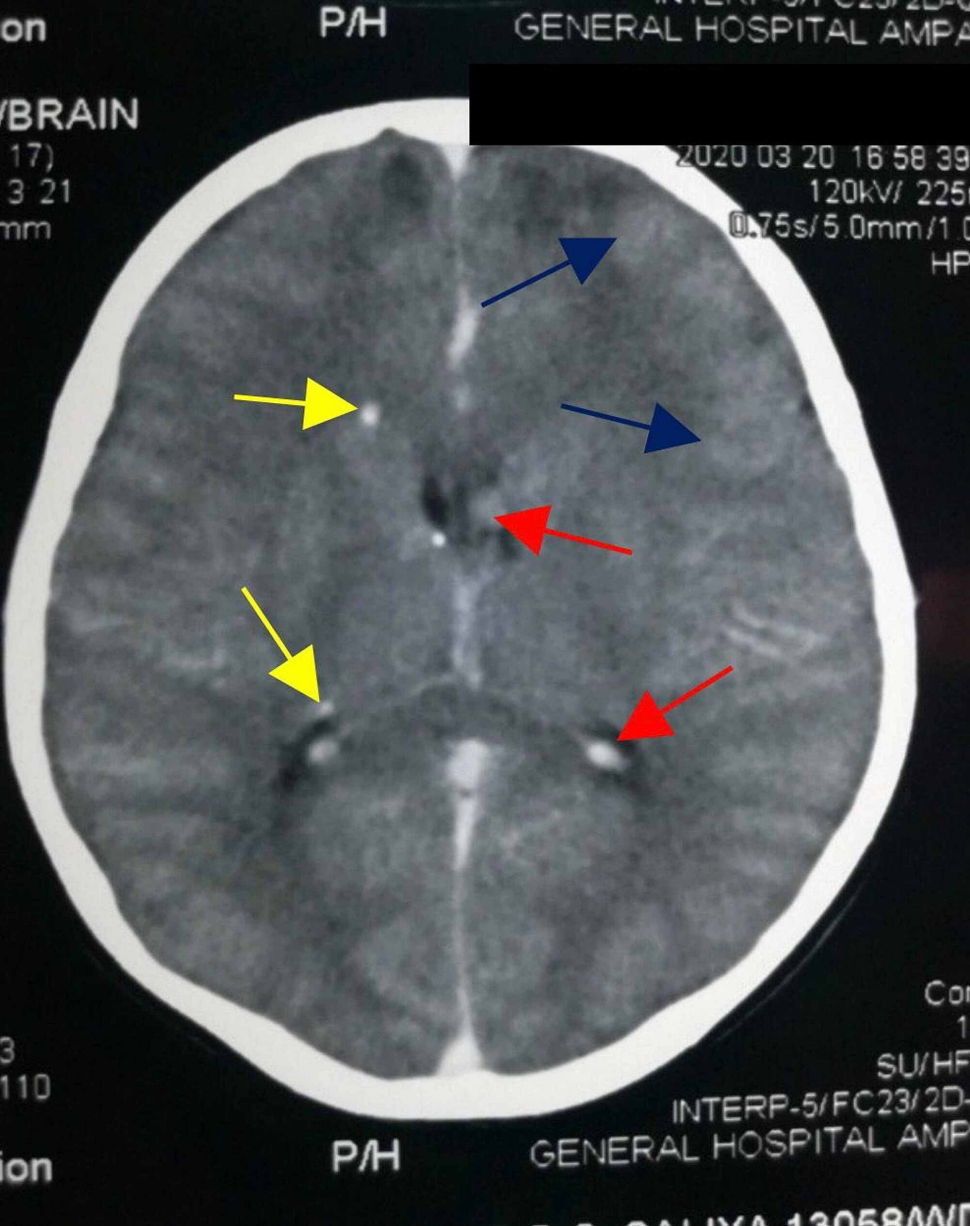
Contrast-enhanced CT showing cortical tubers in bilateral frontal and parietal regions (blue arrows), multiple partially calcified subependymal nodules (red arrows) and coarse calcifications (yellow arrows)

CT brain revealed sub-ependymal giant cell astrocytoma (diameter 12 mm) adjacent to left anterior horn of the lateral ventricle (Figure 4), cortical tubers in bilateral frontal and parietal regions, multiple partially calcified sub-ependymal nodules, and coarse calcifications (Figure 5). Left side retinoscopy revealed a harmatoma; however, no sinister signs were seen. MRI studies were not available for further characterization of brain tumors.

All first-degree relatives were screened based on clinical criteria, and no other family members were found to have criteria fulfilling the diagnosis of tuberous sclerosis. The child was commenced on oral carbamazepine to achieve seizure control and was referred to a pediatric neurologist, neurosurgeon, and psychology team for multi-disciplinary care. Follow-up was arranged to organize serial neuroimaging and clinical monitoring.

## Discussion

Although identification of a mutation in TSC1 or TSC2 itself stands for establishing the diagnosis, genetic diagnosis is not essential, and in poor resource settings, clinical criteria continue to be the main way of diagnosis [6]. The diagnosis of tuberous sclerosis is made in the presence of two major criteria or one major with two minor criteria. The major clinical features include ≥ 3 angiofibromas, shagreen patch, cardiac rhabdomyoma, multiple retinal hamartomas, ≥ 2 ungual fibromas, renal angiomyolipoma, cortical dysplasia including tubers, sub-ependymal nodules, sub-ependymal giant cell astrocytomas (SEGA), hypomelanotic macules, and lymphangioleiomyomatosis. The reported child had five major criteria confirming the clinical diagnosis of tuberous sclerosis.

Sub-ependymal giant cell astrocytomas are seen exclusively in patients with tuberous sclerosis and are found in 10-20% of children with TS during the first two decades of life [7]. The underlying mechanism for these tumors involves the pathogenic activation of mTORC1 [8]. SEGA arises from subependymal nodules, and the histopathology is indistinguishable from sub-ependymal nodules [9]. Whilst these tumors account for 1-2% of all pediatric brain tumors, they carry a high risk for significant morbidity given the potential for obstruction of the drainage of cerebrospinal fluid resulting in hydrocephalus [10]. Parents of children with astrocytoma need to be educated about symptoms of increased intracranial pressure that can arise following acute obstruction and thus, in turn, facilitates them to direct for early neurosurgical interventions. Serial neuroimaging is indicated, and they are likely to demonstrate the increasing size of these nodules that will later grow up to cause SEGA. There are no radiological features described to date that accurately predict the growth potential and risk for developing to SEGA [9].

Early detection of giant cell astrocytoma often enables treatment with medical therapies such as mTOR inhibitors obviating the need for early neurosurgical interventions [11]. Rapamycin (sirolimus) and everolimus are two drugs that have shown efficacy in stabilizing or even shrinking SEGA. Due to immunosuppressant effects, these drugs are contraindicated in patients with severe infections. However, urgent neurosurgical interventions are needed in the presence of life-threatening neurological symptoms, including impending respiratory compromise secondary to the brain stem coning, refractory epilepsy, progressive hydrocephalus, and rapid tumor growth [12]. Stereotactic radiosurgery is also an effective intervention for tumors that do not require immediate neurosurgery [13].

Epilepsy in tuberous sclerosis in the absence of early and appropriate treatment with anti-epileptic drugs has a high probability of progressing to epileptic encephalopathy and cognitive impairment [14]. Most often, epilepsy is diagnosed in children with TS during infancy [15]. Focal seizures and infantile spasms are the most common types of seizures observed in tuberous sclerosis [16]. Whilst vigabatrin is the drug of choice for infantile spasms, focal onset epilepsies can be treated with vigabatrin and other GABA-ergic anti-epileptic drugs and epilepsy surgery [17, 18]. Given the high prevalence of epilepsy and overall poor outcomes of inadequately treated epilepsy in children with TS, it is important that parents are well educated regarding the need for serial monitoring with electroencephalography to diagnose subclinical seizures.

Serial neuroimaging is recommended to all children with TS even if they do not have clinical features for early detection of SEGA [19]. This patient had a relatively late presentation as he did not have any worrying clinical manifestations up to the point he developed focal status. Though he had facial angiofibromas and cutaneous stigmata, parents were not concerned about those features. This report highlights the need for early identification of physical stigmata by both primary care providers and parents in otherwise asymptomatic children with tuberous sclerosis. Early diagnosis and referral are crucial in organizing early multi-disciplinary care to monitor disease progression and prevent complications. Family screening is important as tuberous sclerosis is inherited autosomal dominant and has almost one hundred percent penetrance.

## Conclusions

This report described a child with tuberous sclerosis who had relatively a late presentation and in whom focal status and sub-ependymal tumor were features of the first presentation of tuberous sclerosis. It is crucial that cutaneous stigmata are identified early by parents and primary care providers so that children can be referred for early multi-disciplinary care to monitor the progression of the disease and prevent complications.
